# Artificial gravity as a countermeasure for mitigating physiological deconditioning during long-duration space missions

**DOI:** 10.3389/fnsys.2015.00092

**Published:** 2015-06-17

**Authors:** Gilles R. Clément, Angelia P. Bukley, William H. Paloski

**Affiliations:** ^1^Wyle Science and Engineering GroupHouston, TX, USA; ^2^International Space UniversityArlington, VA, USA; ^3^NASA Johnson Space CenterHouston, TX, USA

**Keywords:** gravity, adaptation, international space station, microgravity, centrifuge, countermeasure

## Abstract

In spite of the experience gained in human space flight since Yuri Gagarin’s historical flight in 1961, there has yet to be identified a completely effective countermeasure for mitigating the effects of weightlessness on humans. Were astronauts to embark upon a journey to Mars today, the 6-month exposure to weightlessness en route would leave them considerably debilitated, even with the implementation of the suite of piece-meal countermeasures currently employed. Continuous or intermittent exposure to simulated gravitational states on board the spacecraft while traveling to and from Mars, also known as artificial gravity, has the potential for enhancing adaptation to Mars gravity and re-adaptation to Earth gravity. Many physiological functions are adversely affected by the weightless environment of spaceflight because they are calibrated for normal, Earth’s gravity. Hence, the concept of artificial gravity is to provide a broad-spectrum replacement for the gravitational forces that naturally occur on the Earth’s surface, thereby avoiding the physiological deconditioning that takes place in weightlessness. Because researchers have long been concerned by the adverse sensorimotor effects that occur in weightlessness as well as in rotating environments, additional study of the complex interactions among sensorimotor and other physiological systems in rotating environments must be undertaken both on Earth and in space before artificial gravity can be implemented.

## Introduction

Preparations for human missions to Mars in the not-too-distant future are underway. As a result of these years long missions, explorers will face severe physiological deconditioning due to weightlessness if new, more effective countermeasures are not developed. Space experiments and operational flight experience have identified detrimental effects on human health and performance as a result of exposure to weightlessness, even when currently available countermeasures are implemented. The requirements for effective countermeasures become more complex as a function of increased mission duration coupled with varying gravity levels as we advance from the International Space Station to the Moon and on to Mars.

Many years of effort on the part of space biomedical engineers have been invested in developing countermeasures to mitigate the physiological deconditioning associated with prolonged weightlessness. Nevertheless, during the first few days after landing, most astronauts experience problems with spatial orientation and balance. Because they risk bone fractures and muscle tears during their recovery period, they must exercise an added degree of caution (Clément, 2011). More effective countermeasures or combinations of countermeasures must be developed because the purpose of a human mission to Mars is to do more than simply survive. Mission success would be greatly compromised if the astronauts arriving at Mars were in no condition to ambulate or carry out basic functions as a result of a weakened physical condition. Sensorimotor performance failures during piloting, extra-vehicular activity, or remote guidance tasks would put the crew at increased risk. Long-duration human missions like going to Mars cannot be seriously considered until the problems associated with weightlessness exposure are successfully addressed.

A number of different countermeasures that are generally aimed at the stimulation of a particular physiological system have been employed in attempts to mitigate the effects of human exposure to weightlessness (Sawin et al., 1998). While for some astronauts these countermeasures are inefficient and onerous, they are reasonably effective against some of the cardiovascular and musculoskeletal losses. However, they have manifested only limited effectiveness in countering the full range of cognitive, sensory, and sensorimotor changes that occur during space flight.

Artificial gravity, in the context of spaceflight, is the simulation of gravity on board a crewed spacecraft achieved by the linear acceleration or steady rotation of all or part of the vehicle (Stone, 1973). Artificial gravity is an alternative approach to addressing the problems of weightlessness-induced effects on the human body. Addressing each individual physiological system in a piecemeal fashion, which is the current mode of operation, is only valid if the principle of superposition holds for the combined effect of these interacting subsystems. All physical and physiological systems are challenged through the application of artificial gravity, which stimulates these systems simultaneously by approximating the normal Earth gravitational environment. Antigravity muscles are activated, bones and the cardiovascular system are stressed, and the otoliths of the vestibular system are stimulated in a manner similar to that on Earth (Young et al., 2006; Clément and Bukley, 2007).

It is obvious that artificial gravity cannot address all of the problems associated with long duration space flight. Clearly it can do nothing for radiation exposure, altered day-night cycles, and the psychological issues that are likely to arise from extended confinement and isolation. What it does is to offer a countermeasure with the potential to address the debilitating and potentially fatal problems of bone loss, cardiovascular deconditioning, muscle weakening, sensorimotor and neurovestibular disturbances, and regulatory disorders. Artificial gravity can be considered as an integrated countermeasure because it addresses all of these systems (Clément and Pavy-Le Traon, 2004).

Another driver for introducing artificial gravity might be as a countermeasure for the impairment of visual acuity and ocular trauma seen in long-duration crewmembers, the so-called *Vision Impairment due to Intracranial Pressure* (VIIP) syndrome. The hypothesized cause for VIIP is that the weightlessness-induced fluid shift is the precipitating factor leading to impairment in cerebrospinal fluid re-sorption and central nervous system venous drainage (Mader et al., 2011). It is possible that interventions like venous limb occlusion and lower body negative pressure could play a preventive role, and these are being investigated. However, continuous or intermittent artificial gravity might be the most efficient countermeasure. For the time being, we do not fully understand all ramifications of the VIIP syndrome and whether it has long-term effects on brain function. However, the VIIP syndrome is currently a matter of great concern for long duration missions.

Many books and review articles have been written on the topic of artificial gravity (Stone, 1973; Lackner and DiZio, 2000; Young et al., 2006; Clément and Bukley, 2007; Hall, 2009). The objective of this paper is to provide a broad overview of *recent* ground-based and in-flight studies that relate to the effects of artificial gravity as a potential countermeasure. One of the major issues associated with humans in rotating environments is the adverse effect on sensorimotor functions. The vestibular system is involved in the regulation of other physiological systems, including respiratory, cardiovascular, circadian, and even bone mineralization systems. The physiological responses of these systems to continuous exposure of humans to anything other than Earth gravity and weightlessness are unknown. Research must be undertaken to identify the minimum level, duration, and frequency of the level of artificial gravity exposure that is needed to maintain normal physiological functions. In addition, the limits for human adaptation to rotation rate, gravity gradient, and Coriolis and cross-coupled accelerations need to be revisited.

## Rotation of the Space Vehicle

**Figure 1:**
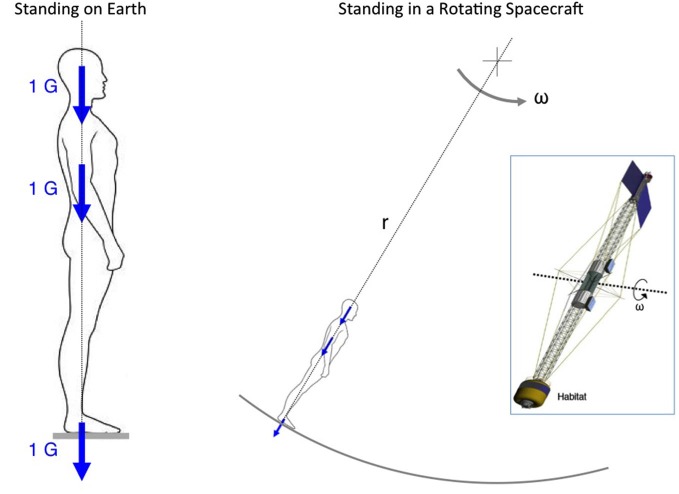
**Artificial gravity**. Continuous rotation of a large spacecraft that creates a centrifugal force of 1 G in the habitat would give the static crewmembers the sensation of standing upright as on Earth. The magnitude of the centrifugal force is function of the square of the rotation rate (ω) times the distance (*r*) from the axis of rotation. In the example of the spacecraft shown in the insert, a 4-rpm rotation rate would generate 1 G in the crew habitat located at 56 m from the axis of rotation.

The rationale for using centrifugation is that the resulting centrifugal force provides an apparent gravity vector during rotation about an eccentric axis. The centrifugal force produced by rotation is a function of the square of the angular rate (ω) and the radius (*r*) of rotation. For example, a crewmember standing on the rim of a habitat rotating at about 4 rpm about an axis located at 56 m would experience the sensation of standing upright closely approximating the same experience as on Earth (Figure 1).

During the early concept phase of human space travel, scientists introduced the idea of creating a substitute for Earth gravity by using centrifugation. Korolev proposed to connect two Voskhod modules by a 300-m tether and rotate them at 1 rpm to produce 0.16 G (Harford, 1973). Inspired by the pioneering works of Oberth (1923) and Noordung (1928), von Braun also proposed a spacecraft having a diameter of 76 m rotating at 3 rpm, the result of which would be a suitable platform for Mars expeditions exposing the occupants to 0.3 G (Von Braun, 1953). On the same principle, O’Neil’s 1.8-km radius Stanford torus spinning at exactly 1 rpm generated Earth gravity to the inhabitants of a space colony (O’Neill, 1977).

When any linear motion is attempted in any plane that is not parallel to the axis of rotation, a Coriolis force is generated. This is a significant drawback associated with rotating environments. The Coriolis force combines with the centrifugal force to produce an apparent gravity vector that differs in magnitude or in both magnitude and direction. This vector may be manifested in two ways for a human walking in a rotating environment: (a) it adds to the apparent weight of the body moving in the direction of rotation and subtracts from the apparent weight when the body is moving in the opposite direction of motion; and (b) when the body moves radially toward the center of rotation, the Coriolis force is exerted at right angles to the body’s motion in the direction of rotation. When the body is moving away from the center of rotation, the force is opposite to the direction of rotation. By contrast, a body motion parallel to the axis of rotation will generate no Coriolis force (Crosbie, 1960; Stone, 1973).

In addition, any angular displacement of the whole body or body part that is not parallel to the spin axis will create cross-coupled angular accelerations that induce stimulation of all three semicircular canals of the vestibular system. Such movement in a stationary environment normally stimulates only the semi-circular canals that correspond to the plane of head rotation. The same head movement in a rotating environment also stimulates the canals that lie in the plane of the rotating environment. The latter combination of canal stimulation results in illusory sensations of bodily or environmental motion and possibly motion sickness (Guedry and Benson, 1978).

Given that centrifugal force depends on both rotation rate and radius, changes in the artificial gravity level can be achieved either by increasing or decreasing the radius, or by increasing or decreasing the rate of rotation. The radius of the structure will have a direct impact on the cost and complexity of the space vehicle, whereas the rotation rate will mostly influence physiological and psychological responses of the crew on board. The final design will be the result of a trade-off study between these two options (Diamandis, 1997).

**Figure 2:**
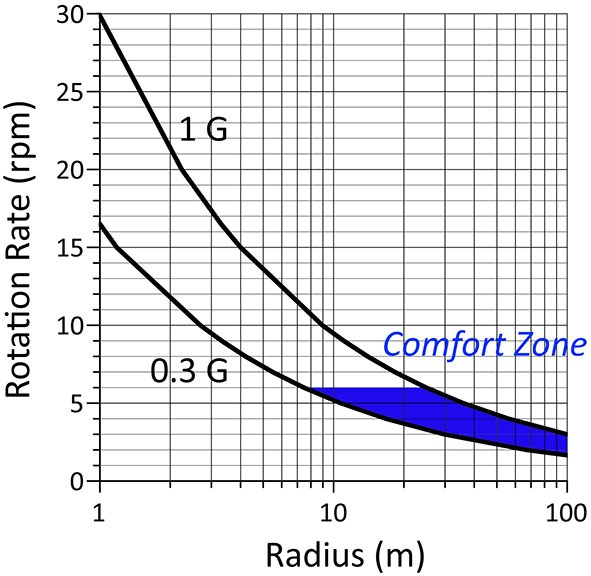
**Hypothetical comfort zone bounded by values of artificial gravity level and rotation rate based on theoretical studies in the 1960s (see Hall, 2009, for details)**. The “comfort zone” is the area in blue delimited by a maximum rotation rate of 6 rpm. According to the model of Stone and Letko (1965) the Coriolis and cross-coupled angular accelerations generated at these rotation rates during walking, climbing and handling materials should be the most comfortable for the crewmembers. However, very little experimental data were actually collected to validate this model. Recent data indicate that the limit of 6 rpm is overly conservative.

The results of studies on humans living aboard slow rotating rooms in the 1960’s (Graybiel et al., 1960, 1965, 1969; Kennedy and Graybiel, 1962; Guedry et al., 1964) suggested that the lightest acceptable system for providing “comfortable” artificial gravity using a rotating spacecraft would be one rotating at 6 rpm at a radius ranging from 12–24 m, such as to create an artificial gravity level ranging from 0.3 G to 1 G (Stone and Letko, 1965; Figure 2). These theoretical limits to rotation rates and radii were based on casual observations of humans walking, climbing, moving objects, and performing nominal head movements in a large-radius centrifuge. These assumptions have largely been taken at face value as correct, but they need to be validated by experimental evidence. More recent data suggest that the adaptation limits of humans to rotating environment are much greater than these earlier studies had anticipated. For example, it has been observed that subjects in a rotating environment could tolerate a rotation rate up to 10 rpm provided that the exposure is progressive (Graybiel et al., 1965) or even up to 23 rpm after habituation of motion sickness symptoms (Young et al., 2001).

Graybiel et al. (1977) noted that when astronauts were making voluntary head movements while being passively spun on a rotating chair on board the Skylab space station, they no longer experienced motion sickness or spatial disorientation after 1 week in orbit. Parabolic flight experiments indicated that “the severity of side effects from Coriolis forces during head movements is gravitational force-dependent, raising the possibility that an artificial gravity level less than 1 G would reduce the motion sickness associated with a given rotation rate” (Lackner and DiZio, 2000). The nausea-inducing effects of Coriolis and cross-coupled accelerations can also be mitigated by restraining head movement during centrifugation. The empirically determined limits for rotation rate and radii proposed in the 60 s for humans to adapt to a rotating environment therefore seem overly conservative. These limits were derived by experimentation under specific limited conditions. Additional experimentation under more extreme conditions may allow extension of these limits. Clearly, further research is warrented.

## Short-Radius Centrifugation

A rotating spacecraft presents serious design, operational, and financial challenges. Practically speaking, it is highly likely that humans do not require gravity (or fraction of it) for 24 h a day 7 days a week to remain healthy. A continuously rotating spacecraft would not be required if intermittent gravity proves to be sufficient. A human rated short-radius centrifuge presents a realistic near-term opportunity for providing intermittent artificial gravity.

A human-rated centrifuge designed for studying vestibular responses to linear accelerations in orbit flew aboard the Space Shuttle Neurolab mission (STS-90) in 1998. This experiment was the first and only in-flight evaluation of artificial gravity on astronauts. The results of this experiment suggested that centrifugal force of 0.5 G and 1 G along the subjects’ longitudinal and transversal axis, respectively, was well tolerated by the crew (Clément et al., 2001). For those astronauts who rode the centrifuge 20 min every other day during a 16-day space mission, cardiovascular deconditioning was reduced (Moore et al., 2005).

A human-powered centrifug that couples exercise with artificial gravity is an interesting and novel approach. Exercise was introduced as a countermeasure in the days of the Gemini program, by means of ingenious elastic, pneumatic, mechanical, hydraulic, and electrical devices. Crewmembers are held “down” by wearing a harness attached to exercise bike or treadmill. Elastic devices only effectively create force, not sustained acceleration. Various designs have been proposed for exercising during centrifugation, such as the “Twin Bike” of the University of Udine (Antonutto et al., 1993; di Prampero, 2000), the “Space Cycle” of the University of California at Irvine (Caiozzo et al., 2004), and NASA Ames Research Center’s human-powered centrifuge (Greenleaf et al., 1996). The assumption is that exercising under such increased inertial forces would decrease the exercise time required to maintain health and fitness in space. If trial results prove positive and the amount of exercise is indeed reduced by centrifugation, such devices are good candidates for long-duration mission countermeasures.

**Figure 3:**
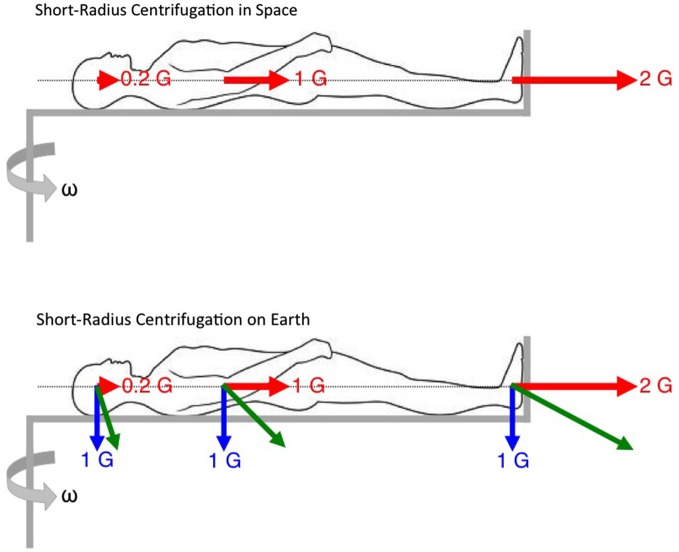
**Constraints for short-radius centrifugation**. On Earth, the actual forces exerted on the body during centrifugation are the resultant of the gravitational force (in blue) and the centrifugal (inertial) forces (in red). These gravito-inertial forces (in green) are larger than 1 G and tilted relative to vertical. In space, the centrifugal forces are the only forces generated by centrifugation and aligned with the longitudinal body axis. Note also the gravity gradient, i.e., the different magnitude of centrifugal force along the longitudinal body axis.

On a short-radius centrifuge, the subjects are generally lying supine with their head close to the axis of rotation and their feet directed outwards. During centrifugation in space, the subject is only exposed to the centrifugal force along their longitudinal body axis, referred to as artificial gravity. However, during centrifugation on Earth, centrifugal force combines with the gravitational force resulting in the so-called gravito-inertial force, which is both larger in magnitude than the centrifugal force itself, and tilted with respect to the longitudinal body axis (Figure 3).

In addition, on a short-radius centrifuge, there is a noticeable difference in the magnitude of centrifugal force at the subject’s head and at the feet. The gravity gradient is the variation in artificial gravity level as a function of distance from the center of rotation. The gravity gradient also has an effect on the hydrostatic pressure along the longitudinal body axis. The hydrostatic pressure influences the circulation of blood to the head and from the lower extremities and therefore affects the functioning of the cardiovascular system. It is not known if the gravity gradient has any critical influences on the cardiovascular and neurovestibular systems.

The Coriolis force is proportional to the linear velocity of the imparted motion, the mass of the moving object, and the rotation rate of the rotating environment. It is important to note that the magnitude of the Coriolis force is not dependent on the radius of the rotating environment. The Coriolis force is therefore equally present in both short- and long-radius centrifuges. For a given centrifugal force level, the rotation rate of an on-board short-radius centrifuge must be greater than that of a rotating spacecraft, so body motion will result in larger Coriolis force. However, the crewmembers’ head, body and limb movements will be more restricted, therefore the crew comfort limits during short-radius centrifugation must be re-evaluated.

Currently unknown and quite logistically difficult to determine is the ideal “prescription” of how much acceleration/gravity is required to maintain normal health and over what period of time. The ideal solution is of course a rotating spacecraft that provides a constant 1 G acceleration. In the case of intermittent centrifugation using an onboard short-radius centrifuge, a research program is needed to identify the gravity levels that are necessary to mitigate the deconditioning of physiological systems, determine how these loads should be applied (e.g., duration, frequency, time of day), and provide protocols to minimize or eliminate undesirable side effects. Artificial gravity should also be integrated with other countermeasures such as exercise, sensorimotor training, and pharmacological prescriptions to optimize crew health.

**Figure 4:**
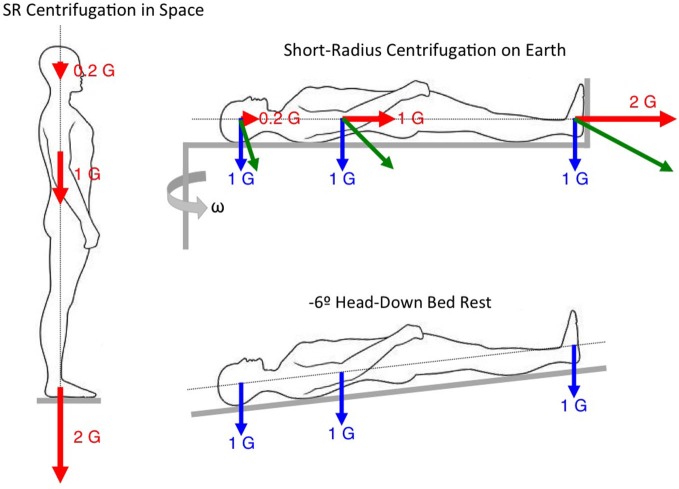
**Rationale for evaluating the effects of intermittent short-radius centrifugation during bed rest**.

One method for evaluating the effects of different levels or duration of gravity loading on the physiological systems is to test whether intermittent short-radius centrifugation can overcome the deconditioning of bed rest. In these investigations, the physiological responses measured during bed rest alone are compared with the same physiological responses during bed rest and intermittent centrifugation. The assumption is that the differences observed between the responses in the two conditions are due to the net forces acting along the longitudinal body axis (Figure 4).

A total of 19 studies have been performed evaluating the effectiveness of short-radius centrifugation providing 1–2 G at the heart during bed rest or dry immersion interventions lasting from 3–28 days. The results of these studies showed that intermittent centrifugation with and without concurrent aerobic exercise during otherwise continuous bed rest: (a) attenuated plasma volume loss (Lee et al., 1997; Iwasaki et al., 1998, 2001, 2005); (b) maintained exercise capacity (Katayama et al., 2004); (c) improved post-bed rest orthostatic tolerance time (Schneider et al., 2002; Iwase, 2005; Watenpaugh et al., 2007; Guinet et al., 2009; Shibata et al., 2010; Linnarsson et al., 2015); and (d) reduced the exaggerated responses to head-up tilt after bed rest, such as elevated heart rate and increased muscle sympathetic nerve activity (Iwasaki et al., 2005).

Very few bed rest and short-radius centrifugation studies have compared bone and muscle changes. Smith et al. (2009) did not find significant differences in bone mineral density during centrifugation compared to controls. However, this study was only 21 days long, whereas traditional countermeasure studies showing changes in bone during bed rest were of much longer duration; e.g., 30 days (Zwart et al., 2007) to 117 days (Shackelford et al., 2004). However, recent 5-day bed rest investigations showed a decrease in serum levels of markers for bone formation (CD200) and an increase in serum levels of markers for bone resorption (CD200R1) in control subjects. In subjects undergoing 30 min of centrifugation per day, the changes in levels of markers for bone homeostasis seen under conditions of bed rest were attenuated, suggesting that centrifugation is a promising countermeasure for bone loss (Kos et al., 2014).

No bed rest studies combined with intermittent centrifugation have examined the structural integrity of muscle fibers (i.e., CSA and distribution by fiber type) after deconditioning, although this test has been performed in many of the traditional countermeasure studies. Future artificial gravity studies on skeletal muscle deconditioning should therefore focus on the analysis of global muscle parameters, like muscle volume and endurance, but also individual muscle fibers by fiber type.

Regarding sensorimotor performance, recent studies have shown that after prolonged bed rest, subjects exhibit significant changes in the monosynaptic stretch reflex and in functional mobility evaluated by the time it takes for subjects to complete an obstacle course (Reschke et al., 2009). However, there were no significant effects of bed rest on the functional stretch reflex and on balance control parameters associated with computerized dynamic posturography (Reschke et al., 2009). Cognitive functioning, assessed by a self-administered battery of tests used on the International Space Station, does not appear to be adversely affected by long-duration head-down bed rest (Seaton et al., 2009). It has been proposed that body unloading long-duration head-down bed rest might serve as an exclusionary analog to differentiate proprioceptive and somatosensory changes from graviceptor changes in post-spaceflight sensorimotor behavior (Reschke et al., 2009). A 21-day bed rest study comparing the neurovestibular effects after bed rest showed no difference in balance control and ocular counter-rolling whether intermittent centrifugation was used or not (Jarchow and Young, 2010). In the same study, the error in the subjective visual vertical was significantly different from zero in the centrifuged group and not different in the control group; however, this effect was short-lived (Moore et al., 2010).

Investigators also tested whether intermittent standing or a combination of heel raising, squatting and hopping exercises was sufficient to prevent alteration in balance and gait following a 5-day bed rest. A cross-over design study was performed with 10 male subjects during 6° head down tilt: (a) with no countermeasure; (b) while standing 25 min per day; (c) during locomotion-like activities 25 min per day. Gait was evaluated by grading subjects’ performance during various locomotion tasks. Equilibrium scores were derived from peak-to-peak anterior-posterior sway while standing on a foam pad with the eyes open or closed or while making pitch head movements. When no countermeasure was used, head movements led to decreased postural stability and increased incidence of falls immediately after bed rest compared to before. When upright standing or locomotion-like exercises were used, postural stability and the incidence of falls were not significantly different from the baseline after bed rest. These results indicate that daily 25 min periods of standing or locomotion-like exercise prove useful against postural instability following a 5-day bed rest. The efficacy of these countermeasures on locomotion could not be evaluated, however, because gait was not found to be altered after a 5-day bed rest (Mulder et al., 2014).

The results of studies using rotating chairs in orbit suggest that the threshold for human perception of verticality is between 0.22 G and 0.5 G (Arrott et al., 1990; Benson et al., 1997; Clément et al., 2001). However, in some investigations the subject’s head was on-center whereas in other studies it was off-center, either on the same side as the feet or on the opposite side (Clément and Reschke, 2008). Recent studies confirmed that the threshold for the perception of verticality in parabolic flight is also comprised between 0.16 G and 0.38 G (de Winkel et al., 2012; Harris et al., 2014). It is interesting to note that this threshold is much higher than the threshold for the perception of linear acceleration, which ranges from 0.005 G to 0.02 G depending on the axis of motion (Benson et al., 1986).

The perception of verticality is also subject-dependent. A recent ground-based study (Clément et al., 2014) attempted to determine the rotation parameters of a short-radius centrifuge so that subjects in the dark would feel as if they were standing upright. Results showed that about half of the subjects felt like they were vertical when centrifugation elicited 1 G at their center of mass along their body longitudinal axis, whereas the other half felt they were vertical when they experienced about 1 G at ear level. Heart rates varied with the subjects’ perception of verticality. These results suggest that one group of subjects was relying principally on the otolith organs for the perception of verticality, whereas the other group was also relying on extra-vestibular somatosensory receptors. The crewmember’s perception of verticality might therefore be a factor to take into account for the prescription for artificial gravity during space flight.

## Partial Gravity Simulators

**Figure 5:**
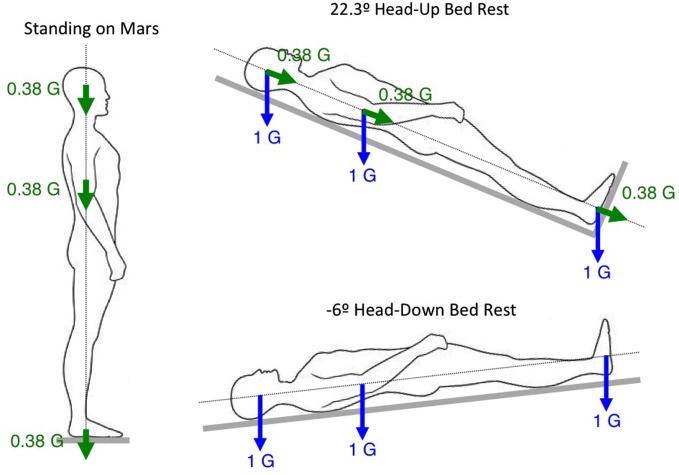
**Rationale for evaluating the effects of Martian gravity during head-up tilt**.

Another method for simulating partial gravity on Earth is through the use of body inclination or suspension techniques. For example, a 9.5° head-up inclination results in a 0.16 G force along the body longitudinal axis, simulating lunar gravity; a 22.3° inclination results in a 0.38 G force along the body longitudinal axis, simulating Martian gravity (Figure 5). The principle seen above for comparing the changes in physiological responses between intermittent centrifugation and bed rest (see Figure 4) can be applied for comparing the changes between static body tilt and bed rest. A previous study used a 9.5° head-up tilt during bed rest to simulate a lunar mission. Subjects were placed in 6° head-down tilt for 4 days to simulate microgravity during the travel to the Moon, then in 9.5° head-up tilt for 6 days to simulate the effects of Moon gravity (0.16 G), and again in 6° head-down tilt for 4 days to simulate the return journey. Muscular exercise was performed during the head-up tilt period to simulate 6 h of lunar EVA. Results showed that hormonal and body fluids responses were not different between this simulated mission and a full 14-day head-down tilt bed rest or a 14-day spaceflight (Pavy-Le Traon et al., 1997). These results indicate that Moon gravity is not effective for preventing cardiovascular deconditioning following spaceflight. It is not known whether Mars gravity is effective or not.

The subjects in these earlier simulations were not weight-bearing, and thus these protocols did not provide an analog for load on the musculoskeletal system. Cavanagh et al. (2013) proposed a novel analog that included the capability to simulate standing and sitting in a lunar loading environment. In the proposed design, the 9.5° head-up tilted bed is mounted on six linear bearings and is free to travel with one degree of freedom along rails, thus allowing approximately 16% weight loading of the feet during standing. They demonstrated that the reaction forces at the feet during periods of standing were a reasonable simulation of lunar standing, and that “lunar” sitting could also be successfully simulated. In fact, during a 6-day test they measured significant changes in the volume of the quadriceps muscles.

A partial gravity environment can also be simulated by unloading a portion of a subject’s weight using suspension techniques. The subjects are either upright or lying on one side. Overhead suspension systems are used to partially or fully unload the subject’s legs by means of cables, springs, and a bicycle harness. This simple set-up provides a satisfactory simulation of a partial gravity environment on the lower extremities. However, the non-vertical lifting force that occurs during locomotion and the discomfort of the harness are the main disadvantages of the system. Also, although the subject experiences less weight, the 1 G force is still acting on their vestibular system and internal organs.

**Figure 6:**
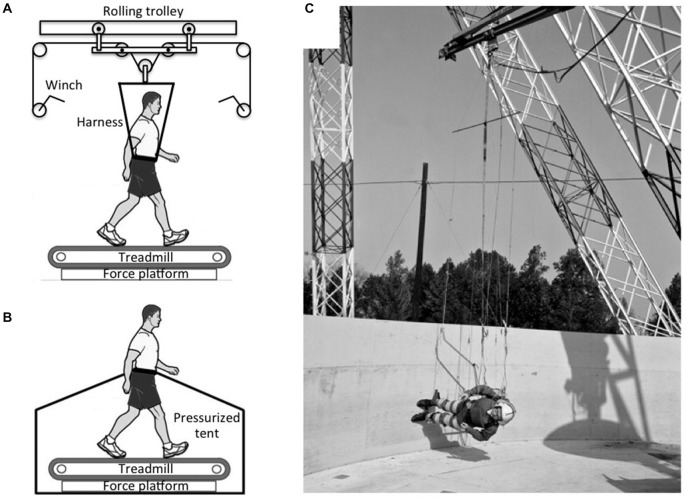
**Partial-gravity simulators. (A)** A harness connected to a rolling-trolley mechanism ensures that only a vertical force is applied to the subject. **(B)** Subject walking on a treadmill with lower body positive pressure (LBPP) support that reduces weight bearing. **(C)** The reduced-gravity walking simulator at NASA Langley Research Center used long cables to support a subject walking on a tilted surface Photo credit: NASA.

The MIT partial gravity simulator, also known as the Moonwalker, is capable of simulating partial gravity as low as 0.05 G (Figure 6A). A recent study tested 12 healthy subjects before and after Martian gravity (0.38 G) simulation to determine the effects of partial gravity adaptation on walking performance. Results showed that the subjects walked with an altered gait characterized by an increased downward center of mass acceleration, reduced muscle activity, and increased maximum joint angles after Martian gravity simulation (Wu, 1999).

Recently, a *lower body positive pressure* (LBPP) treadmill was introduced that offers the ability to study gait dynamics during body weight unloading. While on the treadmill, subjects wear a pair of neoprene shorts with a kayak-style skirt that zips into an air chamber, creating an airtight seal. When the air chamber inflates, there is an increase in air pressure around the lower body that lifts the subject upwards at the hips (Figure 6B), effectively reducing gravitational forces at the feet. The reduction in apparent body weight can range from 1 to 80%. Compared to to a suspension system, the air pressure is applied uniformly over the lower body in the LBPP, thus reducing the formation of pressure points that are common with harness-based systems while maintaining normal muscle activation and gait patterns (Takacs et al., 2013).

Another suspension configuration has the subjects suspended horizontally and allowed to “walk on the wall” of a rotating platform (Figure 6C). The tilt of the walkway determines the magnitude of the force along the subject’s longitudinal body axis. Studies performed with this ingenious system at the NASA Langley Research Center in the 1960s showed that subjects were comfortable walking, running, and jumping at simulated gravity levels ranging from 0.16 to 0.3 G. At levels above 0.3 G, the subjects reported “sensations of leg and body heaviness”, which became quite disturbing at 0.5 G (Letko and Spady, 1970).

A recent study showed that lower body negative pressure combined with walking on a horizontal treadmill for 40 min daily provided enough longitudinal body axial loads to prevent lumbar spine deconditioning following a 28-day bed rest, as this combination produced a force equivalent to one body weight in the head-to-foot direction for a sufficient time period (Macias et al., 2007).

## Research Projects

To help inform the final decision on whether to conduct continuous spin of the whole space vehicle or to intermittently expose the crewmember to short-radius centrifugation, the limits of human adaptation in a rotating environment must be revisited. We need to identify the acceptable and/or optimal ranges for radius and rotation rate to avoid unacceptable crew health and performance consequences. For intermittent applications, we need to identify what level, duration, frequency, and time of day of exposure to artificial gravity are optimal. We also need to investigate the physiological responses to transitions between artificial gravity, microgravity, and Moon or Mars gravity because such studies would be useful in assessing whether dual adaptation to a rotating and a non-rotating environment is possible.

Space agencies are working on a global research program on artificial gravity that would leverage the facilities available around the world (e.g., short- and long-radius centrifuges, slow rotating rooms, bed rest/dry immersion facilities, suspension systems, etc.) and integrate studies on human, animal, and cell models. Standardization of measures performed before and after each artificial gravity intervention will allow for more compatible assessment across various studies. The biomedical measurements will focus on countermeasure validation, medical events, and subject acceptance and comfort.

Regarding sensorimotor performance, artificial gravity projects that could be performed in the near future include the following: (a) test more gravity level values along Gz within the range from microgravity to 1 G, using the methods described above, to reasonably reach conclusions on the threshold, optimal stimulus-response, and saturation for the effects of centrifugation on sensorimotor performance; (b) test the effects of gravity levels higher than 1 G to assess whether increasing the intensity of the Gz stimulus actually reduces the time of exposure needed; (c) compare whether exposure to centrifugation for intermittent, short periods of time in one or multiple sessions is as beneficial as continuous exposure to Earth’s gravity; (d) investigate whether Gz centrifugation reduces intracranial pressure and possibly mitigates the VIIP syndrome; (e) assess whether centrifugation can possibly mitigate post-flight decrease in performance by studying the effect of centrifugation on cognitive and functional tasks; and (f) assess the effects of gravity gradient on spatial orientation by comparing the responses in subjects placed at various distances from the axis of rotation on a long radius centrifuge.

## Conclusion

Although ground-based studies have the potential for determining a sound artificial gravity prescription, validation of these studies can only be performed in space. No human-rated centrifuges that have been built specifically to counteract cardiovascular and musculoskeletal deconditioning have flown in space to date. Some information could be gained from studies using animal models by comparing the potential differences between the effects of an artificial gravity prescription during centrifugation on Earth and in space. However, questions such as what are the impacts of centrifugation inside a space vehicle on the vibration level, motion sickness, or crew time need to be addressed by use of a human-rated centrifuge. The short-term effects of centrifugation could be assessed by studying changes in biomarkers or gene expressions. Any positive results from this space centrifuge would also provide the impetus for further ground-based research.

## Conflict of Interest Statement

The Associate Editor, Ajitkumar Mulavara and the Review Editor, Malcolm Martin Cohen declare that, despite being associated with the National Space Biomedical Research Institute (NSBRI), the review process was handled objectively. The authors declare that the research was conducted in the absence of any commercial or financial relationships that could be construed as a potential conflict of interest.
